# Assessment of individual tumor buds using keratin immunohistochemistry: moderate interobserver agreement suggests a role for machine learning

**DOI:** 10.1038/s41379-019-0434-2

**Published:** 2019-12-16

**Authors:** J. M. Bokhorst, A. Blank, A. Lugli, I. Zlobec, H. Dawson, M. Vieth, L. L. Rijstenberg, S. Brockmoeller, M. Urbanowicz, J. F. Flejou, R. Kirsch, F. Ciompi, J. A. W. M. van der Laak, I. D. Nagtegaal

**Affiliations:** 1 Radboud University Medical Center, Nijmegen, Netherlands; 2 University of Bern, Bern, Switzerland; 3 University of Bayreuth, Bayreuth, Germany; 4 University of Leeds, Leeds, UK; 5 EORTC Translational Research Unit, Brussels, Belgium; 6 Saint-Antoine Hospital, Paris, France; 7 University of Toronto, Toronto, Canada; 8 Center for Medical Image Science and Visualization, Linköping University, Linköping, Sweden

**Keywords:** Prognostic markers, Colon cancer

## Abstract

Tumor budding is a promising and cost-effective biomarker with strong prognostic value in colorectal cancer. However, challenges related to interobserver variability persist. Such variability may be reduced by immunohistochemistry and computer-aided tumor bud selection. Development of computer algorithms for this purpose requires unequivocal examples of individual tumor buds. As such, we undertook a large-scale, international, and digital observer study on individual tumor bud assessment. From a pool of 46 colorectal cancer cases with tumor budding, 3000 tumor bud candidates were selected, largely based on digital image analysis algorithms. For each candidate bud, an image patch (size 256 × 256 µm) was extracted from a pan cytokeratin-stained whole-slide image. Members of an International Tumor Budding Consortium (*n* = 7) were asked to categorize each candidate as either (1) tumor bud, (2) poorly differentiated cluster, or (3) neither, based on current definitions. Agreement was assessed with Cohen’s and Fleiss Kappa statistics. Fleiss Kappa showed moderate overall agreement between observers (0.42 and 0.51), while Cohen’s Kappas ranged from 0.25 to 0.63. Complete agreement by all seven observers was present for only 34% of the 3000 tumor bud candidates, while 59% of the candidates were agreed on by at least five of the seven observers. Despite reports of moderate-to-substantial agreement with respect to tumor budding grade, agreement with respect to individual pan cytokeratin-stained tumor buds is moderate at most. A machine learning approach may prove especially useful for a more robust assessment of individual tumor buds.

## Introduction

Tumor budding, defined as the presence of single cells or small clusters of up to four tumor cells, may be seen at the invasive front of colorectal cancer and other tumor types [1]. Tumor buds (TB) are detached (epithelial) tumor cells that, in close interaction with their microenvironment, transform at least partially into a mesenchymal stem-like phenotype. In the process, TB lose some of their epithelial characteristics and acquire features that are correlated with increased cell motility [2]. TB are morphologically and biologically related to poorly differentiated clusters (PDC’s), which represent larger tumor cell clusters (five or more cells without gland formation), and which are also located at the tumor invasive front [3].

The clinical significance of the extent of TB as an independent risk factor for adverse outcomes in colorectal cancer (CRC) is now well established [4–8]. However, until recently the reporting of tumor budding in routine practice was held back by a lack of internationally accepted criteria and methodology for its assessment. The recent publication of evidence-based recommendations for TB assessment, formulated during the International Tumor Budding Consensus Conference (ITBCC, 2016), removed an important barrier to the wider reporting of TB [1].

As with all adverse histologic features (and especially those requiring quantitation), interobserver agreement of tumor budding is suboptimal. The degree of interobserver variability may be influenced by the type of stain used (H&E vs. immunohistochemistry), the number of output categories, cut-off values, and experience/expertise of the observers [9–11]. Although reported concordance between observers has generally been within acceptable limits, ongoing concerns regarding interobserver variability have continued to hamper clinical adoption. Interobserver variability in TB assessment may be greatly reduced by computer-aided detection systems. A class of algorithms based on deep learning and capable of recognizing complex tissue structures and patterns is emerging as a promising tool in medical imaging and digital pathology, matching, and in some cases surpassing human performance [12–14]. A requirement for successful development of such supervised deep learning algorithms is the availability of a sufficient amount of correctly labeled input data. As such, its application to TB assessment requires a large number of images containing TB which have been confirmed by (a panel of) experienced pathologists.

We have previously assessed tumor budding [15] in digital images from preselected hotspot locations, both on H&E and in cytokeratin 8–18 (CK) stained slides with the purpose of identifying TB for training computer algorithms. Moderate Kappa scores were found between two pathologists in scoring tumor budding grade, and notably few individual buds were annotated by both pathologists (H&E 38% and CK 54% of the total annotated TB), indicating considerable interobserver variation in identifying individual TB.

In the present study, we aim to establish the level of agreement among expert pathologists when assessing a series of 3000 individual tumor bud candidates in digital images of CK stained slides. Our study differs from other observer studies, which have largely focused on overall tumor budding grade rather than individual TB. Digitized images of 3000 TB-candidates were submitted to an international panel of experts, with the request to determine per candidate status as follows: TB, PDC, or neither. We have chosen to derive these TB/PDC candidates mainly from pan-CK stained tissue. For comparison purposes 150 of the 3000 TB-candidates were also offered for evaluation in ITBCC preferred H&E stained version separately.

## Material and methods

### Materials

From three centers, a total of 46 CRC patients were selected, in which routine diagnostic assessment had revealed the presence of TB. Per patient, one paraffin-embedded tissue block was included. Two slides were stained with AE1–AE3 immunohistochemistry at the Dublin University Hospital, five slides were stained with AE1–AE3 immunohistochemistry at Bern University Labs. The remaining 39 slides were stained with H&E, digitally captured (producing whole slide images; WSI), subsequently destained, restained with CK8-18 immunohistochemistry, and scanned again at the Radboud UMC in Nijmegen (procedure described by van den Brand et al. [16]). All slides were scanned with a Pannoramic P250 Flash II scanner (3D-Histech, Hungary) using a 40× objective lens (yielding specimen level pixel-size of 0.24 × 0.24 μm). As only fully anonymized archival tissue was used in this study, the need for ethical approval was waived by the institutional review board of Radboud UMC.

The set of 3000 TB-candidates was comprised of 800 TB-candidates, taken from a previous study [15]. The remaining 2200 TB-candidates were selected as follows. One pathologist marked hotspot regions (measuring 0.785 mm^2^) in the immunohistochemistry stained slides. Next, an image analysis algorithm was used to automatically identify individual bud candidates. The algorithm performed a color deconvolution [17] to isolate DAB positive image pixels, which were grouped to form binary objects. Only objects with a surface area between 25 and 5000 μm^2^ were retained, to remove small artifacts and larger clusters of tumor cells. Candidates were visually checked by an expert and obvious artifacts were omitted. From the remaining objects, 1900 TB-candidates with surface area <1000 μm^2^ were taken (mostly representing TB) and another 300 candidates with surface area >1000 μm^2^ (containing mainly PDC’s) were randomly chosen, to ensure sufficient presence of TB. The set of 3000 candidates was randomly split into two groups of 1500 cases.

Restaining of the slides allowed us to select identical objects in both H&E and CK immunohistochemistry stains. A part of the 3000 candidates was traced based on their immunohistochemistry image coordinates into the equivalent images in H&E stain. A total of 150 TB-candidates were sampled in H&E, selecting these randomly from different groups (i.e., TB, PDC, neither) in proportion to the number of objects per group.

## Method

**Fig. 1:**
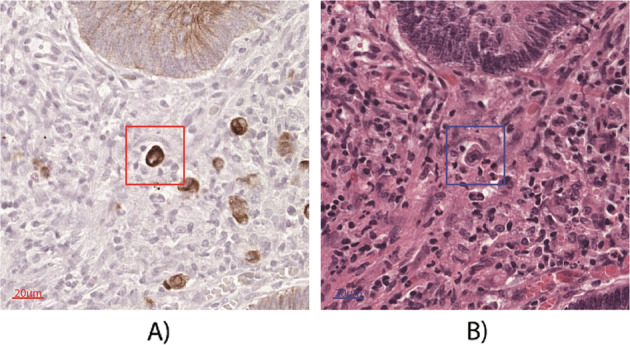
Example of TB-candidate in IHC and re-stained H&E. Example of TB-candidate in **a** immunohistochemistry, and **b** restained H&E.

Eleven pathologists from seven different institutions across seven different countries, all with expertise in the field of tumor budding, were invited to participate in this study. Pathologists were asked to evaluate two study sets each including 1500 TB/PDC candidates in immunohistochemistry and, 2 months later, a set of 150 TB/PDC candidates on H&E according to ITBCC guidelines and best practice, including the rule to regard a visible presence of the nucleus as a requirement. Observers were asked to designate the candidates into one of three categories, namely (1) TB, (2) PDC, or (3) neither (no TB, no PDC). The “gold standard” status of an object was defined using a voting rule of 70% or more: the label is assigned if at least 5 out of 7 and 8 out of 11 pathologists agree in immunohistochemistry and H&E, respectively. To enable simultaneous execution of these tasks by different pathologists from the various work locations, a web-based platform was used. Through this platform, patches of 0.25 × 0.25 mm were shown, based on the center of mass (CoM) of each candidate. To clearly mark the object of interest, a square with a fixed size of 0.03 mm^2^ was added, based again on its CoM. An example is shown in Fig. 1.

Fleiss Kappa and Cohen’s Kappa were used to calculate agreement between multiple observers and different combinations of two observers (one vs. one) respectively. For the Kappa scores we used the terms formulated by Landis and Koch [18], i.e., <0.2 poor, 0.21–0.40 fair, 0.40–0.60 moderate, 0.61–0.80 good, and >0.80 very good.

## Results

Immunohistochemistry results were obtained from nine pathologists, five of whom scored both sets of 1500 TB/PDC candidates. The remaining four pathologists scored either the first or the second 1500 candidates. As a result, 7 scores were obtained per TB/PDC object.

**Fig. 2:**
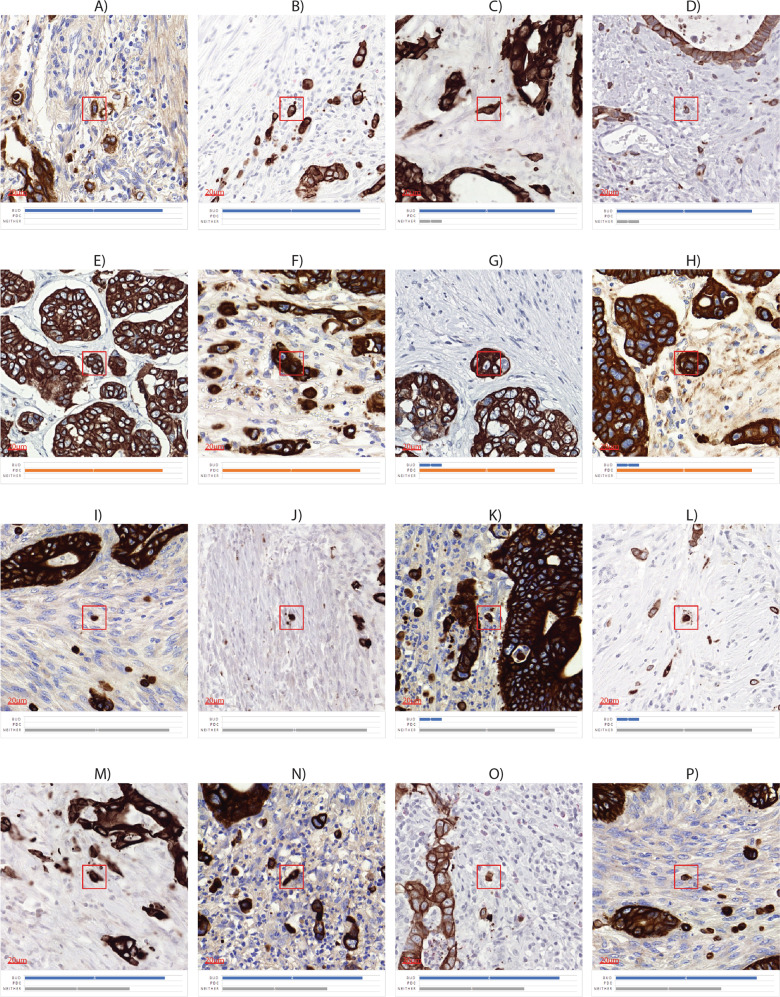
Examples of TB-candidates with manual scores. Examples of TB-candidates **a**, **b** uniform selected as bud, **c**, **d** majority vote bud, **e**, **f** uniform PDC, **g**, **h** majority vote PDC, **i**, **j** uniform neither, **k**, **l** majority vote neither. **m**–**p** no agreement was reached. Legend of colors: blue—bud, orange—PDC, gray—neither.

Of the 3000 TB/PDC candidates, 612, 15, and 386 cases were unanimously classified as TB positive, PDC positive, or neither, respectively. Based on the previously agreed 70%-majority vote, we came to a further definite classification for 1765 candidates (1010 TB, 52 PDC, and 703 neither). For the remaining 1235 candidates, no consensus could be achieved. An overview of all scores can be found in Table 1; examples of every category can be found in Fig. 2.

**Table 1:** Number of objects, classified as TB, PDC, or neither by all, majority, or minority (no agreement) per candidate group (3000 IHC, 150 H&E, and 150 IHC).

	Uniform			70% majority			No agreement (all classes)
	Bud	PDC	Neither	Bud	PDC	Neither	
IHC (3000 objects)	612 (20.40%)	15 (0.50%)	386 (12.87%)	398 (13.27%)	37 (1.23%)	317 (10.57%)	1235 (41.17%)
H&E (150 objects)	8 (5.33%)	1 (0.67%)	13 (8.67%)	31 (20.67%)	10 (6.67%)	68 (45.33%)	19 (12.67%)
IHC reference group (150 objects)	26 (17.33%)	5 (3.33%)	25 (16.00%)	25 (16.67%)	7 (4.67%)	24 (16.00%)	38 (25.33%)

**Fig. 3:**
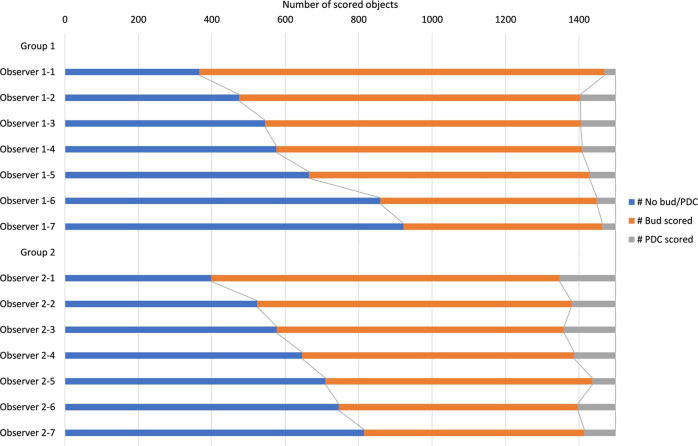
TB, PDC and neither scores in the 2 × 1500 immunohistochemistry dataset per observer.

Of the 2 × 1500 immunohistochemistry candidates, on average 803 (±194), 67 (±28), and 630 (±201) were classified as TB, PDC, or neither in Group 1. In Group 2, 758 (±118), 111 (±31), and 631 (±143) candidates were labeled as TB, PDC, or neither (Table 2). Individual scores are shown in Fig. 3.

**Table 2:** Averaged number of objects (with std. dev.) per observer group.

Per observer group × dataset: Mean number (std. dev.) of TB, PDC, or neither			
	Bud	PDC	Neither
IHC—Group 1 (1500 objects)	803 (193)	67 (28)	630 (201)
IHC—Group 2 (1500 objects)	758 (118)	111 (30)	631 (142)
H&E (150 objects)	54 (18)	13 (2)	84 (19)
IHC reference group (150 objects)	70 (14)	12 (2)	68 (14)

Fleiss Kappa was calculated for the first 1500 TB-candidates (classification result Group 1) and the second 1500 TB-candidates (classification result Group 2) separately. With values of 0.42 for the first group and 0.51 for the second group, assessment in both groups showed moderate agreement.

**Fig. 4:**
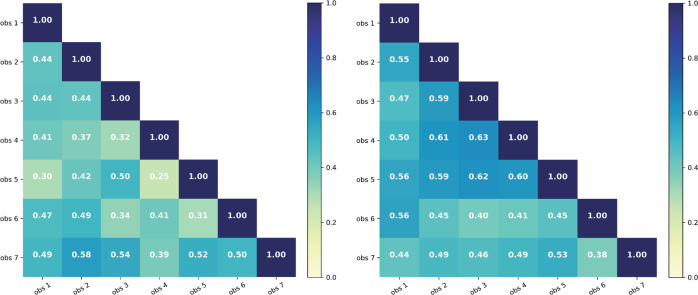
One-versus-one Cohen Kappa scores per observer of **left** group 1 and **right** group 2.

One-versus-one observer agreement numbers (Cohen's Kappa) are shown per group in Fig. 4. In the first group these scores vary between 0.24 and 0.60, in the second group they range from 0.36 to 0.65. In the second observer group, the 2nd, 3rd, 4th, and 5th observer achieved a higher agreement score compared with each other. This is also reflected in the lower standard deviation numbers of the second group compared with the first one.

**Fig. 5:**
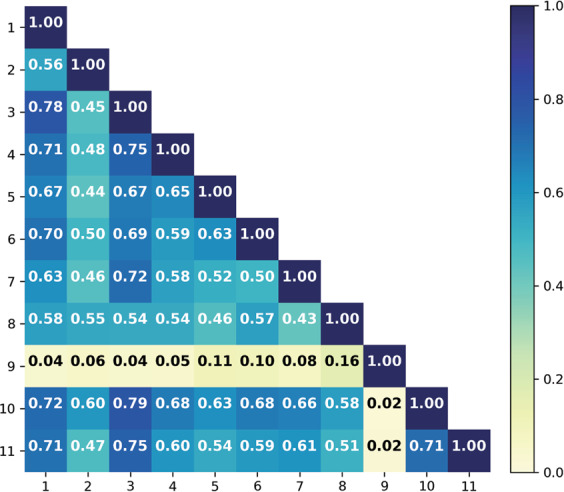
Cohen Kappa scores per observer for the 150 H&E cases.

**Fig. 6:**
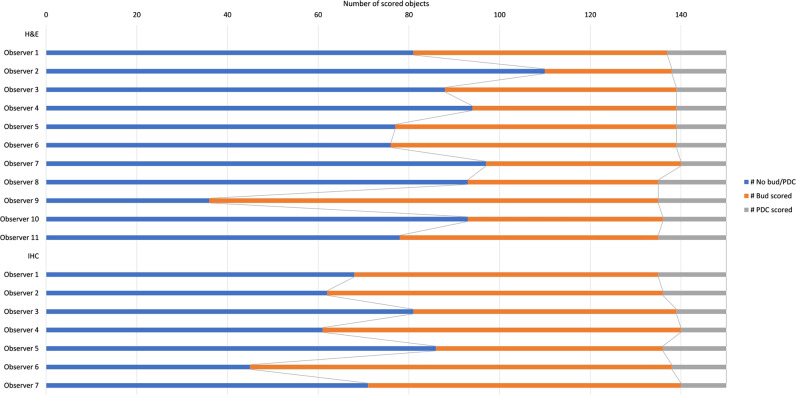
TB, PDC, and neither scores in the 150 H&E dataset per observer with the addition of scores of the identical objects in immunohistochemistry staining.

A series of 150 TB/PDC candidates on H&E stained sections was assessed by 11 observers. A Fleiss Kappa of 0.49 was achieved on this subset. The Cohen Kappa numbers ranged from 0.07 to 0.782 (Fig. 5). Of the 150 H&E candidates on average 54 (±18), 13 (±2), and 84 (±19), were classified as TB, PDC, or neither. Individual scores can be found in Fig. 6.

Table 1 shows that 8, 1, and 13 cases were unanimously classified as TB positive, PDC positive, and neither respectively. Based on the majority vote we counted 39 TB, 11 PDC, and 81 neither. On 19 cases no agreement could be reached.

**Fig. 7:**
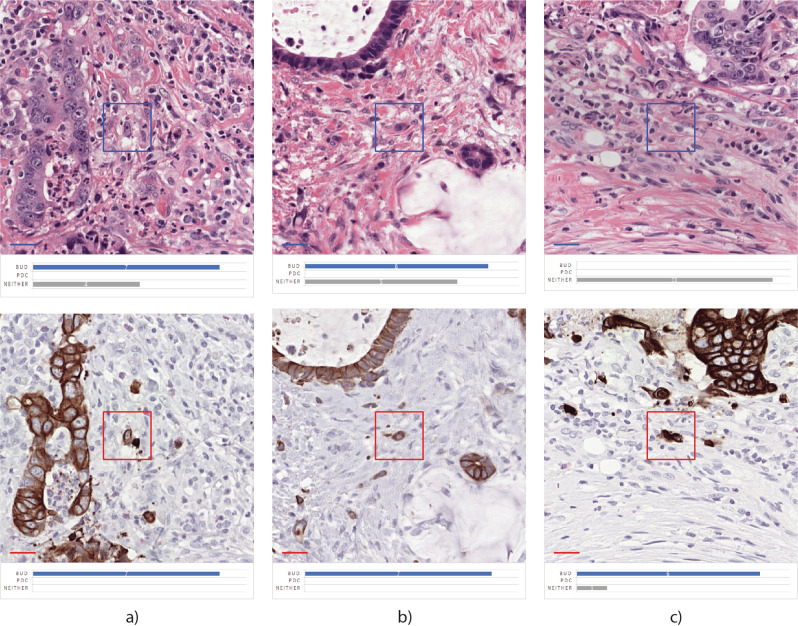
Score shifted examples of restained TB-candidates. **a**, **b** Shift in the agreement from uniform bud vote in immunohistochemistry to no agreement in H&E, **c** shift from majority vote on tumor bud in immunohistochemistry to uniform neither assessment in H&E.

The number of “no agreement” objects was therefore decreased in H&E compared with the immunohistochemistry reference group. Numbers of PDC remained virtually unchanged, but the number of objects, classified as TB by at least 5 of the 7 observers (Uniform + Majority), decreased on balance with higher numbers of neither class (25 + 24 versus 13 + 68 objects). Table 2 also shows that the average number of PDCs in the H&E group almost corresponds to the average number of PDCs in the immunohistochemistry reference group. Example cases can be found in Fig. 7.

## Discussion

In contrast to previous studies, we assessed observer variability in scoring individual TB, rather than the overall budding. This approach prevented pathologists from unsubconsciously taking the tumor grade and differentiation grade into account when assessing individual TB. Accurate identification of individual TB is critical for subsequent development of artificial intelligence models which can automate this task, thereby potentially reducing observer bias. We found that the agreement between pathologists for scoring buds in immunohistochemistry was only moderate, which was slightly improved if buds were identified in H&E. Only one in three of the 3000 immunohistochemically stained candidates was uniformly classified as either TB, PDC, or neither by unanimity or majority vote. No agreement was reached on average for four out of ten candidates. Since interobserver variability in the assessment of individual TB can result in variability in tumor budding grades, it is of interest to investigate the cause(s) of such variability.

In the interpretation and assessment of structures in immunohistochemistry, TB/PDCs can be mistaken for other cytokeratin-positive objects. These objects can be partially described as remnants, parts of epithelium that is at some stage of degradation. Various studies provide examples of this. Mitrovic et al. [19] cites fragmentation of neoplastic glandular structures as a possible cause of misclassification. In line with this, Kai et al. [20] call the presence of “pseudo buds” an unexpected pitfall in the assessment of TB in immunohistochemistry, especially in cases with severe inflammation. They consider pseudo buds to be comprised of fragmented epithelium, destroyed by inflammatory cells. Koelzer et al. [9] note that the assessment of TB in immunohistochemistry can be complicated by the presence of cytokeratin-positive microvesicles and membrane fragments. Although not mentioned, degenerating or apoptotic cells are also part of this CK positive group.

In contrast, Shinto et al. [21] researched the morphology, configuration, and role of small nonnucleated cytoplasmic fragments (minimum diameter 2 μm) often detected around TB. The authors observe that some cytoplasmic fragments are clearly connected with budding foci, suggesting that they represent dendritic cell processes, rather than isolated cell fragments. They, therefore, have renamed them cytoplasmic pseudo fragments.

As we used a minimum area of 25 μm^2^ for the computer-aided preselection of TB-candidates, cytoplasmic pseudo fragments were removed a priori from the candidate group. As a consequence, interobserver variability can only be associated with the first-mentioned objects in this study. Since no definite cause could be found in the candidates for whom no agreement was reached, further research will have to show whether/to what extent each of them actually contributes to interobserver disagreement.

Clear presence of a cell nucleus is an important criterion for TB/PDC as it allows observers to distinguish TB/PDC from aforementioned CK positives.

Although CK staining helps in the first phase of detection of the (malignant) epithelium, e.g., the hotspot selection, slight overstaining can be counterproductive because informative variations in intensity that help the interpretation of morphological structures can be lost for the human observer. It is therefore possible that part of the disagreement in immunohistochemistry can be traced to poor visibility of the nuclei in this study, where observers did not have much opportunity to fall back on relevant information from the context, i.e., nearby gland fully intact or not, position of the candidate relative to the gland, but also frame of reference for estimating area/size of the candidate.

As is well known, the human eye is quite poor at judging minor variations in intensity, but computers are especially good at assessing finer shades of intensity. A number of researchers have now entered the field of automatic detection of TB in IHC stained slides. In a recent article, Bergler et al. [22] present a hybrid method. In a first phase detection step, TB-candidates are generated using classical image processing methods, whereby the segmentation of pan-CK positive objects primarily is performed on color information or signal intensity thresholds and information on size. A second operation then is added to this phase, which is aimed at reducing the number of false positives and carried out with the help of deep learning-based algorithms. Weis et al. [23] follow a similar protocol for immunohistochemistry stained TMA cores. Both authors show promising results but need further validation before implementation in daily clinical routine.

When H&E sections were presented, a group of 11 observers achieved the same moderate overall performance on the assessment of 150 TB/PDC candidates. The interobserver agreement on H&E is similar to the level of agreement on immunohistochemistry. We did see a shift in the classifications, however, there was a decrease in candidates classified as TB and an increase in candidates classified as neither. The variability in classification of TB’s on H&E—other than in immunohistochemistry—may be related to challenges in differentiating TB from inflammatory cells (lymphocytes, histiocytes, and macrophages) and stromal cells, as activated fibroblasts [19]. Based on this observation, we hypothesize that interobserver variability may be less attributable to TB composed of clusters of 2–4 cells than to individual cell TB. As the number of cells of the candidates was not registered, we could not test this hypothesis in this study. It is worth considering this in future investigations.

Relevant morphological cell characteristics are better preserved in H&E, but TB detection is then more time-consuming. Furthermore, there is a greater chance of TB’s being overlooked compared to IHC, where the main issue is “overcall” due to the presence of TB mimics. Nevertheless, based on the results of this study, there is no reason to deviate from the ITBCC preference for H&E staining at this time. It should be noted, however, that only a relatively small number of TB-candidates were classified in H&E sections.

In conclusion, we have shown that the assessment of individual TB-candidates in immunohistochemistry is difficult, even for pathologists with expertise in the field of TB. Although immunohistochemical staining helps with the detection of TB/PDC candidates, there is a risk of misclassification in connection with the common presence of cytokeratin-positive “competitors”. The assessment of these objects must be done correctly, on the basis of nuclear absence. Doubtful (in-)visibility of the cell nucleus in immunohistochemistry is in this context a complicating factor that can be remedied with the use of computers.

In this study we have already applied a form of computer-aided selection by automated preselection based on color and size. With the results obtained, further steps can now be taken to achieve automation of TB assessment.
