# Correction to: Assessment of individual tumor buds using keratin immunohistochemistry: moderate interobserver agreement suggests a role for machine learning

**DOI:** 10.1038/s41379-019-0450-2

**Published:** 2020-01-03

**Authors:** J. M. Bokhorst, A. Blank, A. Lugli, I. Zlobec, H. Dawson, M. Vieth, L. L. Rijstenberg, S. Brockmoeller, M. Urbanowicz, J. F. Flejou, R. Kirsch, F. Ciompi, J. A. W. M. van der Laak, I. D. Nagtegaal

**Affiliations:** 1 Radboud University Medical Center, Nijmegen, Netherlands; 2 University of Bern, Bern, Switzerland; 3 University of Bayreuth, Bayreuth, Germany; 4 University of Leeds, Leeds, UK; 5 EORTC Translational Research Unit, Brussels, Belgium; 6 Saint-Antoine Hospital, Paris, France; 7 University of Toronto, Toronto, Canada; 8 Center for Medical Image Science and Visualization, Linköping University, Linköping, Sweden

**Keywords:** Prognostic markers, Colon cancer

**Correction to: Modern Pathology**

10.1038/s41379-019-0434-2

The article Assessment of individual tumor buds using keratin immunohistochemistry: moderate interobserver agreement suggests a role for machine learning, written by J. M. Bokhorst, A. Blank, A. Lugli, I. Zlobec, H. Dawson, M. Vieth, L. L. Rijstenberg, S. Brockmoeller, M. Urbanowicz, J. F. Flejou, R. Kirsch, F. Ciompi, J. A. W. M. van der Laak and I. D. Nagtegaal, was originally published electronically on the publisher’s internet portal on 16^th^ December 2019 without open access. With the authors’ decision to opt for Open Choice the copyright of the article changed on 3rd January 2020 to © The Author(s) 2019 and the article is forthwith distributed under a Creative Commons Attribution 4.0 International License (https://creativecommons.org/licenses/by/4.0/), which permits use, sharing, adaptation, distribution and reproduction in any medium or format, as long as you give appropriate credit to the original author(s) and the source, provide a link to the Creative Commons licence, and indicate if changes were made.

